# Lipidomics and machine learning revealing dysregulation of specific triacylglycerol and phosphatidylglycerol as hub lipids associated with fetal growth in gestational diabetes mellitus

**DOI:** 10.7717/peerj.20841

**Published:** 2026-04-07

**Authors:** Zhenguo Lin, Lei Li, Yuhang Fan, Hao Chen, Yanhua Cai, Rongrong Yang, Mei Chen, Guolin Hong

**Affiliations:** 1 Department of Clinical Medicine, Xiamen Medical College, Xiamen, China; 2 School of Public Health, Xiamen University, Xiamen, China; 3 Department of Basic Medical Science, Xiamen Medical College, Xiamen, China; 4 The First Affiliated Hospital of Xiamen University, Xiamen, China

**Keywords:** Gestational diabetes mellitus, Lipidomics, Biomarkers, LogitBoost, Fetal growth

## Abstract

**Objective:**

Emerging evidence links lipid metabolism to the pathogenesis of gestational diabetes mellitus (GDM). This study aimed to identify lipidomic biomarkers and explore their clinical significance for GDM and related fetal growth and development through serum lipid profiling.

**Methods:**

Lipidomic profiles of pregnant women with and without GDM were analyzed using Orthogonal Partial Least Squares Discriminant Analysis (OPLS-DA), Uniform Manifold Approximation and Projection (UMAP), volcano plots, and heatmaps. Carbon chain length and unsaturation effects on fold change (FC) were evaluated. Pathway analysis was performed *via* the Lipid Ontology (LION) platform, while lipid networks were constructed using Debiased Sparse Partial Correlation (DSPC). Hub lipids were identified through topological analysis and visualized with UpsetR. A GDM detection model was developed using Boruta and LogitBoost algorithms, assessed by receiver operating characteristic (ROC) curve analysis, and interpreted *via* Local Interpretable Model-agnostic Explanations (LIME).

**Results:**

Twelve serum lipid metabolites were significantly associated with GDM risk. Phosphatidylglycerol (PG)(O-27:1) and triacylglycerol (TG)(35.5) were identified as hub lipids. The GDM detection model, incorporating TG(35:5), PG(O-27:1), total protein (TP), and red blood cell distribution width (RDW), achieved high accuracy.

**Conclusion:**

This study preliminarily characterized lipid metabolic pathway disturbances in patients with GDM, highlighting the potential of integrating lipidomics with interpretable machine learning techniques for biomarker discovery and mechanistic insight.

## Introduction

Gestational diabetes mellitus (GDM), a prevalent metabolic disorder in pregnant women, has emerged as a significant public health concern due to the obesity and diabetes epidemic. It affects around 20 million pregnancies globally each year (Ali et al., 2022; Johns et al., 2018), leading to short- and long-term adverse health outcomes for both mothers and children, such as pre-eclampsia, macrosomia, shoulder dystocia, perinatal hypoglycemia, respiratory distress, and an increased risk of type 2 diabetes mellitus, hypertension, and cardiovascular disease later in life (Metzger, 2007; Bellamy et al., 2009). However, the etiology of GDM remains elusive.

Although GDM is characterized by impaired glucose metabolism, GDM has also raised concerns about the role of abnormal lipid metabolism in the pathogenesis of the disease (White et al., 2017; Bao et al., 2018). Some studies have shown that pregnancy with GDM has lipid metabolism disorders, including elevated total triacylglycerol (TG), lower high-density lipoprotein cholesterol (HDL-C) in specific size, and higher low-density lipoprotein cholesterol (LDL-C) and very low-density lipoprotein cholesterol (VLDL-C) (Pinto et al., 2015; Mokkala et al., 2020). For instance, a prospective nested case-control study conducted in Chinese women revealed that the plasma lipidomic profiles in early pregnancy were significantly associated with the subsequent risk of developing GDM (Wang et al., 2021). Traditional clinical lipid indicators or their ratios have been widely discussed, but as suggested by the research exploring the association between maternal TGs and disturbed glucose metabolism in pregnancy, which highlights the mediating role of these factors in the link between pre-pregnancy overweight/obesity and macrosomia (Eppel et al., 2021; Song et al., 2022), they fail to fully reflect the complexity of the altered lipid metabolism associated with GDM (Ryckman et al., 2015; Li et al., 2015; Hu et al., 2021). Lipidomics provides a high-throughput approach to comprehensively assess the types of small molecule lipids in biological samples, helping us to understand the molecular mechanisms involved in the occurrence of GDM.

In recent years, machine learning (ML) techniques have been widely used in disease diagnosis with the development of methodologies, but previous studies have mainly just focused on model construction (Mayerle et al., 2018; Beam & Kohane, 2018). ML algorithms are characterized by the ability to take omics data with high dimensionality into account, which may provide opportunities for selecting the critical lipids for disease detection.

Accordingly, this study was designed to develop and apply an interpretable machine-learning framework to identify potential lipid biomarkers linked to GDM and to elucidate their underlying molecular mechanisms. In parallel, we aimed to examine the associations between these candidate biomarkers and fetal biometry parameters, thereby offering preliminary insights into how maternal lipid metabolism might influence fetal growth. The results of this work as a pilot study are expected to lay a foundation for subsequent large-scale validation studies on the topic.

## Subjects and Methods

### Study population and power analysis

Pregnant women receiving routine antenatal care and delivering at the First Affiliated Hospital of Xiamen University between May and December 2022 were enrolled. Inclusion criteria: (1) singleton pregnancy; (2) maternal age 18–45 years; (3) no pre-pregnancy history of diabetes, hypertension, thyroid disorders, or cardiovascular/cerebrovascular diseases; (4) complete questionnaire and health record data. At 24–27 weeks of gestation, participants completed interviewer-administered questionnaires, underwent a 75 g oral glucose tolerance test (OGTT), and provided fasting blood samples. Diagnosis of GDM based on the International Association of Diabetes and Pregnancy Study Groups (IADPSG) criteria, as adopted by the American Diabetes Association in their 2011 update (Weinert, 2010), was defined by fasting, 1-hour, and 2-hour plasma glucose thresholds of ≥5.1, ≥10.0, and ≥8.5 mmol/L, respectively. Initially, each group of GDM and non-GDM recruited 150 participants. After application of strict inclusion criteria, 51 GDM and 52 non-GDM participants meeting the inclusion criteria provided analyzable fasting blood samples.

Power analysis was performed, under the false discovery rate (FDR) controlled two-sample framework designed for omics data (Liu & Hwang, 2007). With the achieved sample size of the total 103 subjects, we derived a common standard deviation *σ* = 0.846, Cohen’s *d* = 0.61, and a conservative *π*_0_ = 0.50. These parameters yielded an estimated power of 85% (83–87% across *π*_0_ = 0.45–0.55), confirming that our existing study provided adequate statistical power for detecting the anticipated effect size while controlling FDR at 5%.

### Medical data collection and measurement

Baseline information on maternal sociodemographic characteristics and lifestyle was collected through a structured questionnaire. Reproductive factors, medical history characteristics, and fetal biometric parameters on growth by ultrasonography were extracted from the maternal health system. The results of the biochemical tests, the routine blood test and the OGTT were also collected. In addition, maternal sleep quality and depressive mood were assessed using the Pittsburgh Sleep Quality Index (PSQI) and the Edinburgh Postnatal Depression Scale (EPDS), respectively (Cox, Holden & Sagovsky, 1987; Buysse et al., 1989).

### Lipid extraction and quantitative lipidomic profiling

Fasting blood samples (≥8 h) were drawn and stored at −80 °C. Lipid extraction was performed in accordance with a previously published standardized method (Zhang et al., 2021; Klassen et al., 2021). For the controlled thawing of blood serum samples, we transferred the samples from a −80 °C ultra-low temperature freezer to a −20 °C freezer, and then to a 4 °C refrigerator until they were completely thawed. The lipid extraction procedure was as follows. (1) In a 12.0 mL pre-calcined glass test tube sterilized at 400 °C, 80 µL serum was mixed with 10 µL internal standards SPLASH LipidoMIX™ (Avanti, Lot #330707-01-011). Subsequently, 600 µL methanol, 1,000 µL chloroform, and 500 µL ultrapure water were added, vortexed for 15 s between each, and incubated at room temperature for 10 min before centrifugation at 4,000 rpm at 4 °C for 10 min to separate phases. (2) The lower organic phase was then transferred to a fresh 12 mL glass test tube for a secondary extraction with 600 µL chloroform. The combined organic phases were evaporated under a nitrogen stream to dryness. (3) Reconstituted with 100 µL of a 50:50 (v/v) methanol-to-isopropanol mixture, the dried lipid extracts were centrifuged at 4000 rpm and 4 °C, and the supernatant was transferred to a 200 µL insert tube for injection. (4) A quality control (QC) sample, prepared by pooling 10 µL of supernatant from 25 redissolved samples, was essential for assessing the reproducibility and stability of lipidomics analysis. Additionally, a 50% methanol blank solution was utilized to evaluate and minimize potential background interference.

Lipidomic analysis was conducted using Dionex HPLC and Q-Exactive Orbitrap MS. The mobile phases consisted of phase A (acetonitrile/Milli-Q water, volume/volume: 60/40) and phase B (isopropanol/acetonitrile, volume/volume: 90/10). Data-independent acquisition mode was employed for analysis. A 2 microliter injection volume and a flow rate of 0.6 mL/min were used with a 25-minute gradient elution program. The MS setup comprised a 4.3 kV heated electrospray ionization source scanning 250–1,200 m/z, with probe heater and capillary temperatures at 325 °C and 330 °C, respectively. Sheath gas and auxiliary gas flow rates were set to 35 and 12. Randomized sample injection and periodic QC samples ensured process reliability and stability.

### Data analysis and bioinformatic analysis

Data analysis and bioinformatics were conducted using R (version 3.6.3), MetaboAnalyst (version 6.0.0), and Cytoscape (version 3.8.0). Statistical significance was assessed *via* Wilcoxon or Chi-square tests, with a *p*-value < 0.05 indicating significance. At the same time, in order to render our research results more rigorous and scientific, we used the principal component analysis (PCA) method to evaluate the potential confounding bias effects brought about by demographic variables and sociological variables (Khan et al., 2019). By placing the lipidome together with the “PSQI score”, “EPDS score”, “production”, “age”, and “education level”, the latter five original variables as confounders, and conducting PCA on the whole of variables mentioned above, we determined the representative mixed principal components of the confounding variables based on the variance contribution rates and the principal component loadings. Regarding the mixed principal components as the independent variable and the groups as the dependent variable, we further analyzed whether the representative principal components of the confounding variables had a statistically significant confounding effect on the study objective by the logistic regression method. In the Orthogonal Partial Least Squares Discriminant Analysis (OPLS-DA) model, lipids with variable importance in the projection (VIP) > 1, fold change (FC) > 1.5 or FC < 0.67 (equivalent to |logFC| > 0.58) and FDR < 0.05 were identified as differential metabolites. Uniform Manifold Approximation and Projection (UMAP) was utilized to dimensionally reduce features while retaining data intrinsics. Volcano plot and heatmap were used for differential expression visualization. Spearman’s rank correlation analysis was used to examine how fetal ultrasonographic parameters and maternal gestational status related to the lipid profiles. Carbon chain length-logFC and unsaturation-logFC trend analysis characterized lipid traits on risk of GDM. Differential lipids were subjected to Lipid Ontology (LION) analysis, wherein lipid species were mapped onto the LION hierarchy to annotate the enrichment of specific functional classes and infer systematic alterations in lipid metabolism. The Debiased Sparse Partial Correlation (DSPC) method constructed the lipid network graph, with eight topological methods—Closeness, Degree, Density of Maximum Neighborhood Component (DMNC), Edge Percolated Component (EPC), Maximal Clique Centrality (MCC), Maximum Neighborhood Component (MNC), Radiality, and Stress—used to identify hub lipids and UpsetR (Upset plot) visualized these results. The Boruta, feature selection method, selected lipids and clinical laboratory indicators based on feature importance. When the GDM detection model optimal hyperparameter (value of iterations) had been determined by the 3-repeated 10-fold cross-validation, the study dataset was partitioned into training and validation subsets internally. And a panel of the aforementioned indicators was fed into LogitBoost algorithm to build the GDM detection model whose performance was assessed by the area under curve (AUC) based on the curve of receiver operating characteristic (ROC). Local Interpretable Model-agnostic Explanations (LIME) provided an interpretable framework for the combined indicators.

### Ethical approval

This study was approved by the Ethics Committee of the First Affiliated Hospital of Xiamen University (2020-No.050) and written informed consent was obtained from all study participants.

## Results

### Baseline characteristics of the study participants

**Figure 1:**
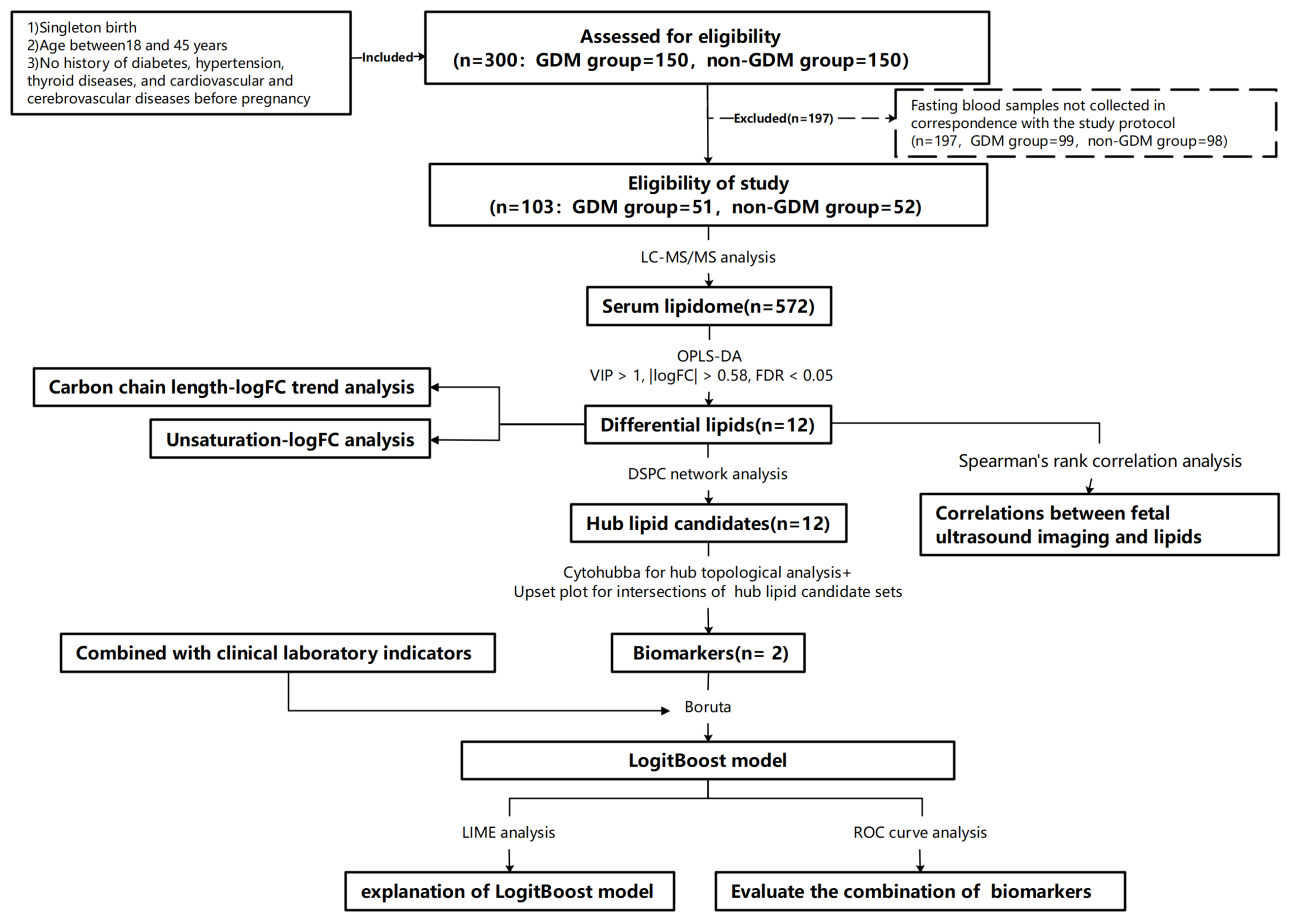
The flowchart showing the process of lipidomic analysis integrated with machine learning and statistical analysis in the study.

In this study, 51 cases in the GDM group and 52 cases in the non-GDM group were selected for analysis. Figure 1 showed the process of lipidomic analysis integrated with machine learning and statistical analysis in the study. Table 1 presents the baseline characteristics of the participants, which indicated that there were no statistically significant differences between the GDM group and the non-GDM group in terms of age, parity, educational attainment, sleep quality, and the total score of the EPDS.

**Table 1:** Baseline characteristics of study participants.

Baseline variables	GDM (*n* = 51)	Non-GDM (*n* = 52)	*p*-value
Age, years	32.00 (30.00, 36.0)	32.50 (30.00, 35.00)	0.814
Parity, n (%)			0.628
Not less than 1 born child	24 (47.06%)	21 (40.38%)	
None of born child	27 (52.94%)	31 (59.62%)	
Education level, n (%)			0.818
Above college or others	44 (86.27%)	43 (82.69%)	
Below senior high school	7 (13.73%)	9 (17.31%)	
Sleep quality (PSQI), n (%)			0.620
Good sleep quality (PSQI≤5)	28 (54.90%)	25 (48.08%)	
Not bad and worse (PSQI>5)	23 (45.10%)	27 (51.92%)	
Depressive status, n (%)			0.999
With depressive mood (EPDS score≥10)	7 (13.73%)	8 (15.38%)	
Without depressive mood (EPDS <10)	44 (86.27%)	44 (84.62%)	
Total TG	2.24 (1.69, 2.86)	2.02 (1.68, 2.57)	0.470
LDL-C	2.96 (2.30, 3.52)	2.89 (2.19, 3.49)	0.678
HDL-C	2.25 (1.91, 2.71)	2.01 (1.84, 2.42)	0.189
Pre-pregnancy BMI	22.27 (20.77, 23.62)	21.68 (19.87, 24.12)	0.243

**Notes.**

Data were reported in Q2 (Q1, Q3) or frequency (%).

### Evaluation of biases from confounders and preliminary screening of lipids

Based on participants’ serum samples, lipidomic profiling quantified 572 lipids classified according to the LIPID Metabolites and Pathways Strategy (http://www.lipidmaps.org). Then, during the process of evaluating biases, a total of 11 principal components were extracted from all the variables included in PCA. Among them, the mixed principal component 1 and the mixed principal component 2 chiefly captured lipid metabolite variance, accounting for 24.10% and 16.14% of the total variance contribution, respectively, both exceeding 10%. However, no mixed principal component emerged whose loadings predominantly indexed the confounders. Mixed principal component 1 was the statistically significant influencing factor for the groups (*p*-value < 0.05), indicating that the impact of biases from confounders on the study was slight and acceptable. In subsequent research, we employed OPLS-DA to conduct an in-depth analysis of lipid samples. Utilizing the criteria for preliminary screening of lipids, we successfully identified 12 lipids with the highest statistical significance. Corresponding results were presented in Table 2.

**Table 2:** Association of lipid species with the risk of GDM.

Differential lipids	VIP value	FC value	FDR value
LPC(22:3)	1.89	0.641	0.00649
PG(O-27:1)	1.96	0.616	0.00649
TG(35:5)	1.71	0.526	0.00663
LPC(O-14:0)	1.97	0.592	0.00663
PE(O-47:5)	1.95	0.641	0.00775
TG(P-33:3)	1.61	0.559	0.00941
TG(O-33:4)	1.54	0.583	0.00989
TG(33:6COOH)	1.93	0.526	0.0118
TG(44:4CHO)	1.02	0.586	0.0205
TG(29:0)	1.56	0.511	0.0205
PI(O-42:5)	1.07	1.56	0.0256
LPC(24:5)	1.39	0.599	0.0380

**Notes.**

FDR values were determined using Wilcoxon test.

Abbreviations LPC lysophosphatidylcholine PI phosphatidylinositol PE phosphatidylethanolamine TG triacylglycerol PG phosphatidylglycerol

### Visualization of lipidomic profiles

**Figure 2:**
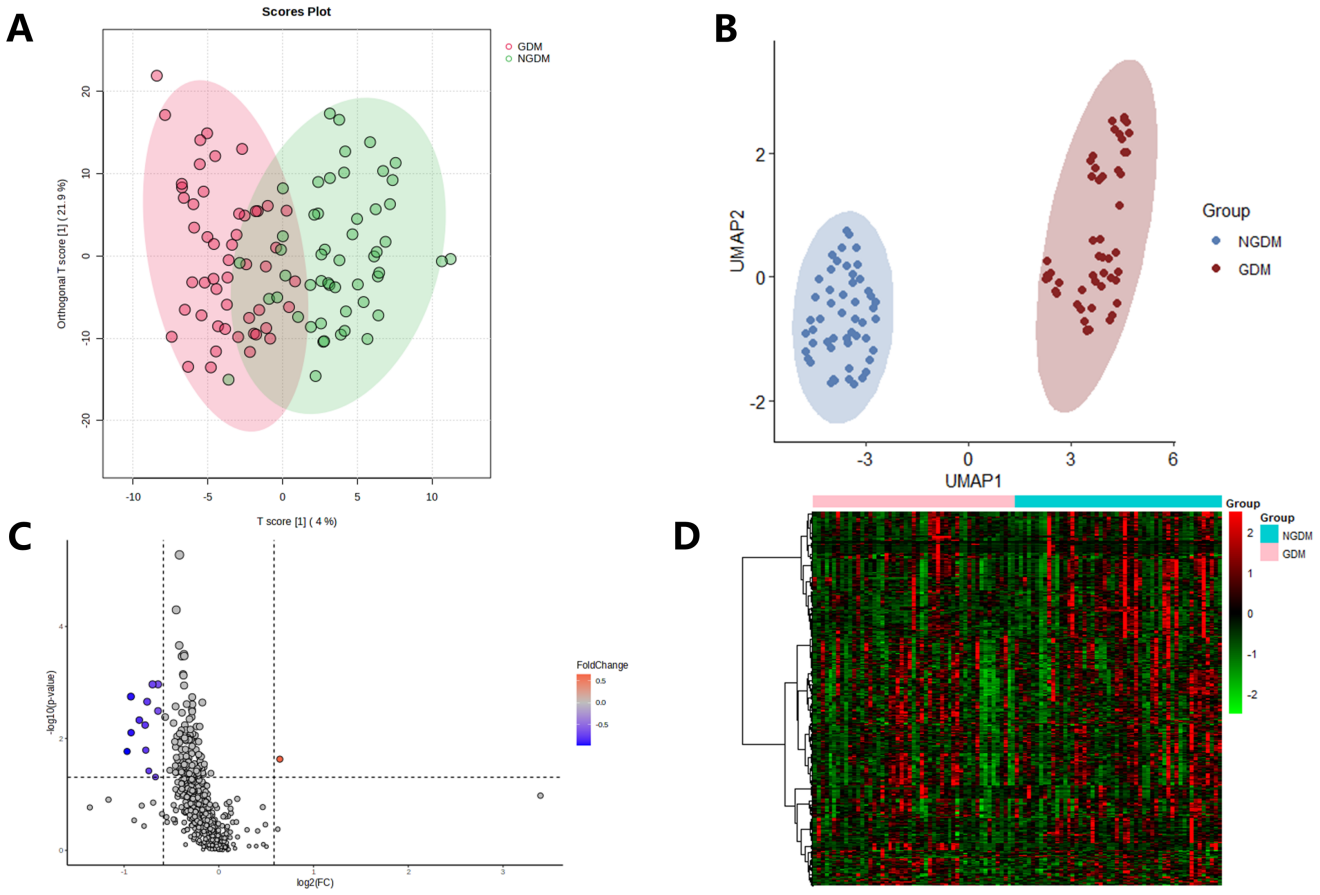
Comparative analysis of lipidomic profiles between GDM and non-GDM groups. (A) The participants from GDM group (red) and non-GDM group (green) in the OPLS-DA score plot. (B) The participants from GDM group (reddish-brown) and non-GDM group (blue) in the UMAP plot. (C) The volcano plot of lipids: grey, blue and red respectively indicating no significant difference in lipids between the two groups, significant downregulation and upregulation. (D) The heatmap with hierarchical clustering: rows representing the lipids, columns representing the samples of the participants, green and red representing lower and higher expression levels, respectively.

The resulting score plot of OPLS-DA showed a relative difference between the lipid profiles of the GDM and non-GDM groups (R2Y = 0.677, Q2Y = 0.263; Fig. 2A). And the permutation test confirmed the robustness of the OPLS-DA model (*p*-value for R2Y < 0.05 and Q2Y < 0.001). As displayed in the UMAP plots, GDM group was not overlapped with non-GDM group (Fig. 2B). In the volcano plot there were 12 differential lipids, 1 lipid up-regulated and 11 lipids down-regulated (Fig. 2C). The lipid level patterns in GDM group and non-GDM group by heatmap, were shown in Fig. 2D.

Furthermore, LION enrichment analysis of differential lipids resulted in the top significant terms including “fatty acid with 3–5 double bonds” and “positive intrinsic curvature” (Table 3).

**Table 3:** Lipid enrichment analysis highlighting significant differences in lipid profiles.

Term ID	Discription	Annotated total lipids	Significant lipids	Expected value		*p*-value
LION:0002977	Fatty acid with 3–5 double bonds	7	LPC(22:3)	0.15	0.008
			LPC(24:5)		
			LPC(22:3)		
LION:0000466	Positive intrinsic curvature	44	LPC(24:5)	0.92	0.0096
			LPC(O-14:0)		
			PI(O-42:5)		

**Notes.**

LION enrichment analysis of significant differential lipids was shown.

### Correlation analysis of fetal ultrasonographic parameters and maternal gestational status with the lipid profiles

**Figure 3:**
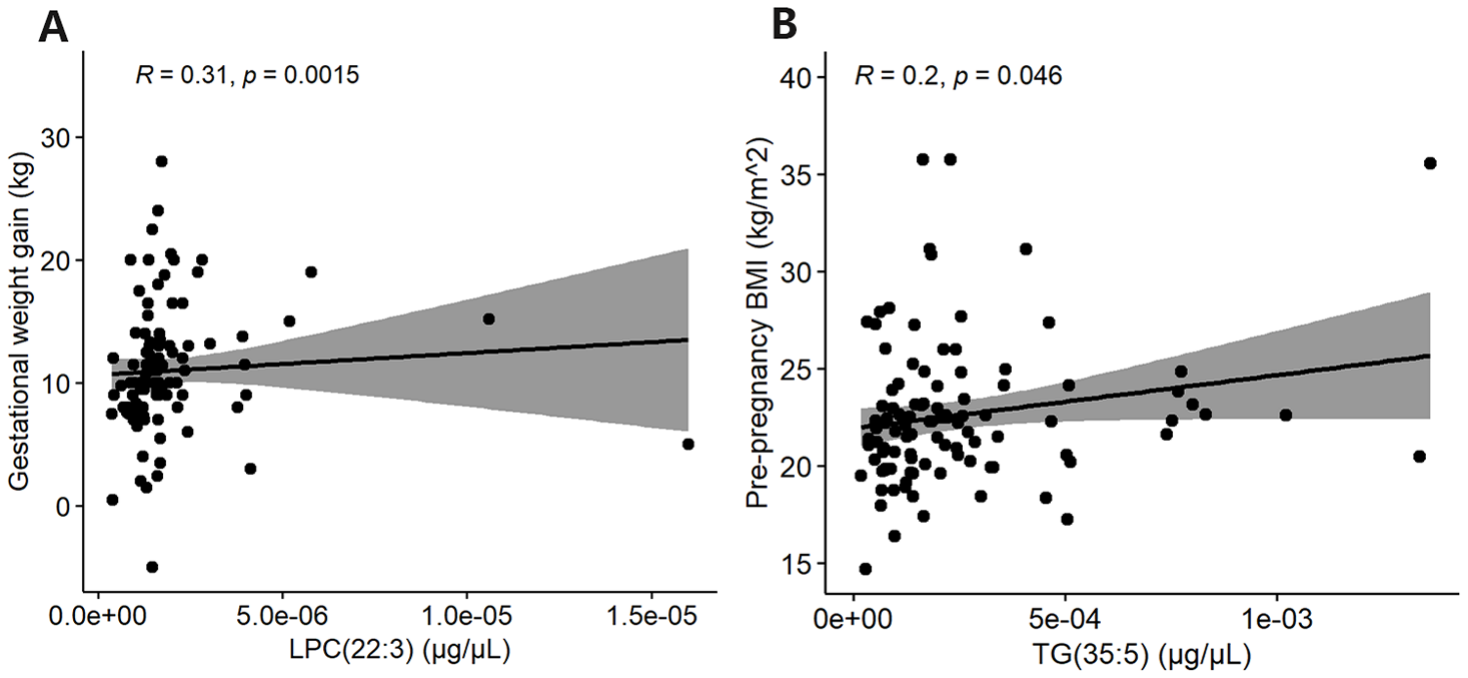
Spearman’s rank correlation analysis of lipids in maternal serum and maternal gestational status. (A) The scatterplot reflecting correlation between LPC(22:3) and gestational weight gain. (B) The scatterplot reflecting correlation between TG(35:5) and pre-pregnancy BMI.

**Figure 4:**
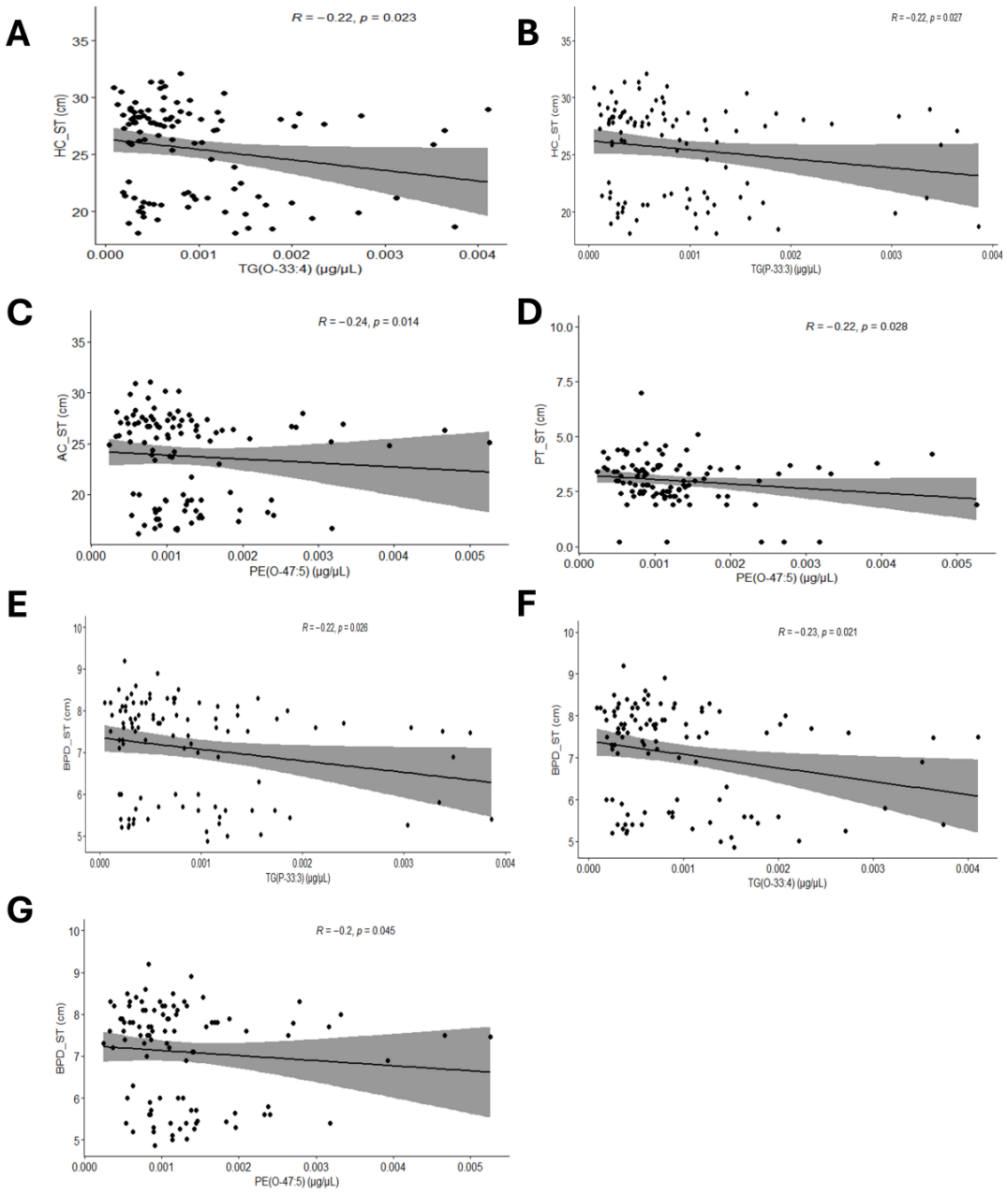
Spearman’s rank correlation analysis of lipids in maternal serum and mid-gestation fetal growth indicators. (A) The scatterplot reflecting correlation between TG(O-33:4) and HC_ST. (B) The scatterplot reflecting correlation between TG(P-33:3) and HC_ST. (C) The scatterplot reflecting correlation between PE(O-47:5) and AC_ST. (D) The scatterplot reflecting correlation between PE(O-47:5) and PT_ST. (E) The scatterplot reflecting correlation between TG(P-33:3) and BPD_ST. (F) The scatterplot reflecting correlation between TG(O-33:4) and BPD_ST. (G) The scatterplot reflecting correlation between PE(O-47:5) and BPD_ST.

**Figure 5:**
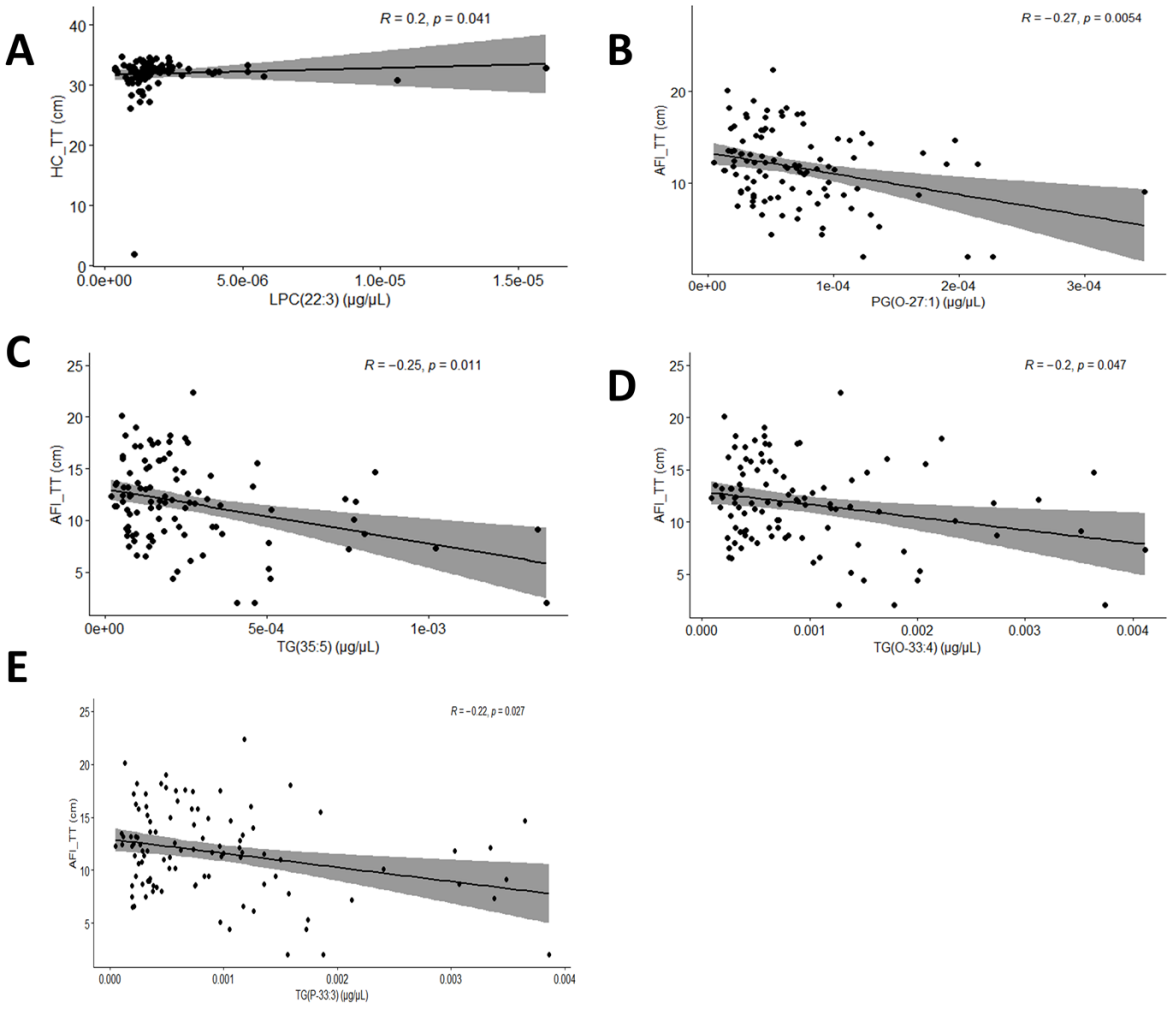
Spearman’s rank correlation analysis of lipids in maternal serum and late-gestation fetal growth indicators. (A) The scatterplot reflecting correlation between LPC(22:3) and HC_TT. (B) The scatterplot reflecting correlation between PG(O-27:1) and AFI_TT. (C) The scatterplot reflecting correlation between TG(35:5) and AFI_TT. (D) The scatterplot reflecting correlation between TG(O-33:4) and AFI_TT. (E) The scatterplot reflecting correlation between TG(P-33:3) and AFI_TT.

In aspect of the relationships between maternal gestational status and lipid profiles, gestational weight gain rose in tandem with lysophosphatidylcholine (LPC) (22:3) while body mass index (BMI) advanced in step with TG(35:5) (Figs. 3A, 3B). In aspect of the relationships between fetal ultrasonographic parameters and lipid profiles, our observations of mid-term pregnancy revealed there were two pairs of negative correlations: TG(O-33:4) and fetal head circumference (HC_ST), TG(P-33:3) and HC_ST (Figs. 4A, 4B). Furthermore, phosphatidylethanolamine (PE) (O-47:5) exhibited a negative correlation not only with fetal abdominal circumference (AC_ST) but also with fetal placental thickness (PT_ST) (Figs. 4C, 4D). Additionally, TG(P-33:3), TG(O-33:4) and PE(O-47:5) were all found to be negatively correlated with fetal biparietal diameter (BPD_ST) (Figs. 4E, 4F, 4G). Subsequently, upon integrating serum lipid profiles with late-gestation fetal growth indicators, a positive correlation was identified between LPC(22:3) and fetal head circumference (HC_TT) (Fig. 5A). PG(O-27:1), TG(35:5), TG(O-33:4) and TG(P-33:3) were negatively correlated with amniotic fluid index (AFI_TT) (Figs. 5B, 5C, 5D, 5E).

### Overview of logFC variation trends in lipid species

**Figure 6:**
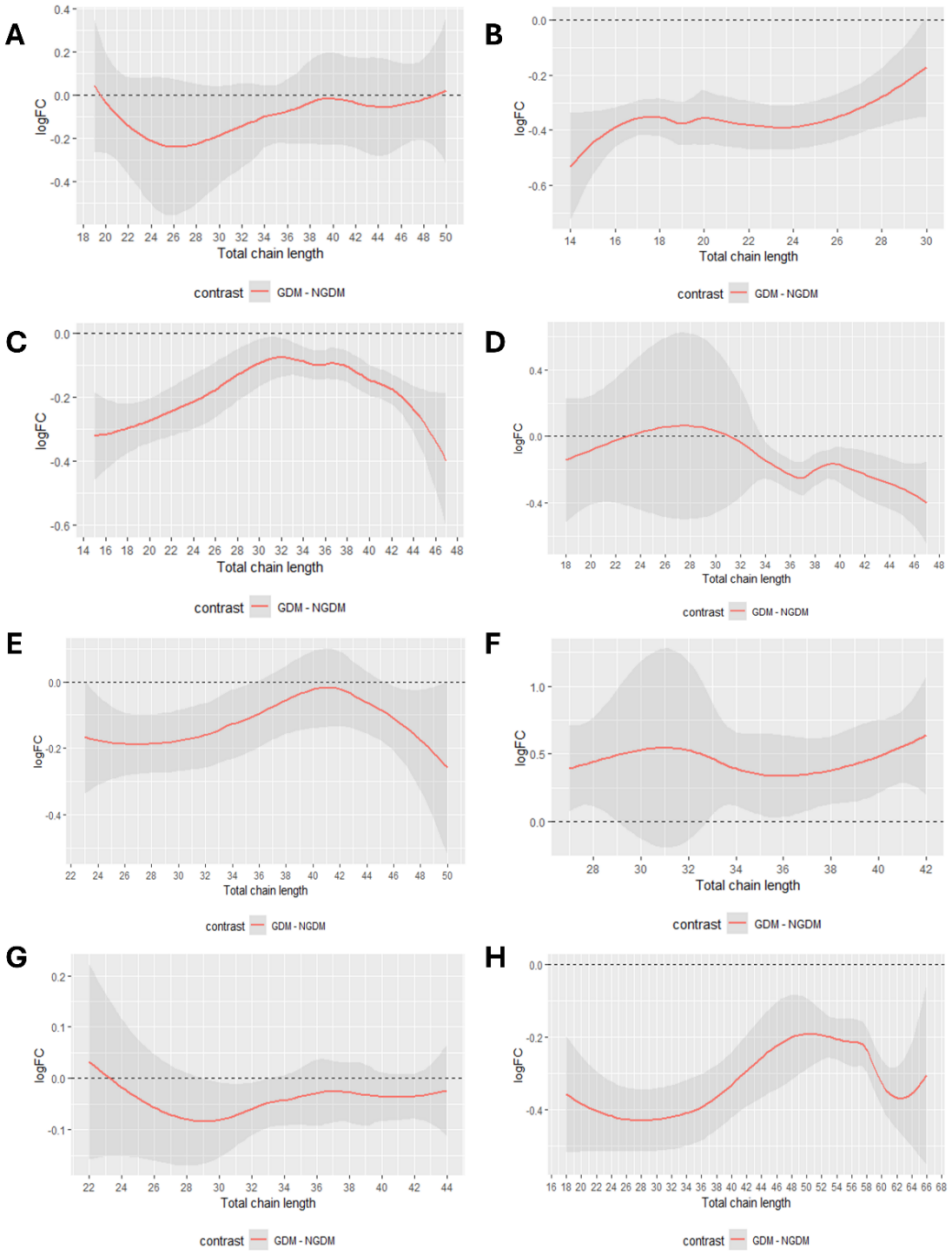
Analysis of carbon chain length-logFC in lipids. (A) The total chain length trend analysis plot of DG. (B) The total chain length trend analysis plot of LPC. (C) The total chain length trend analysis plot of PC. (D) The total chain length trend analysis plot of PE. (E) The total chain length trend analysis plot of PG. (F) The total chain length trend analysis plot of PI. (G) The total chain length trend analysis plot of SM. (H) The total chain length trend analysis plot of TG.

**Figure 7:**
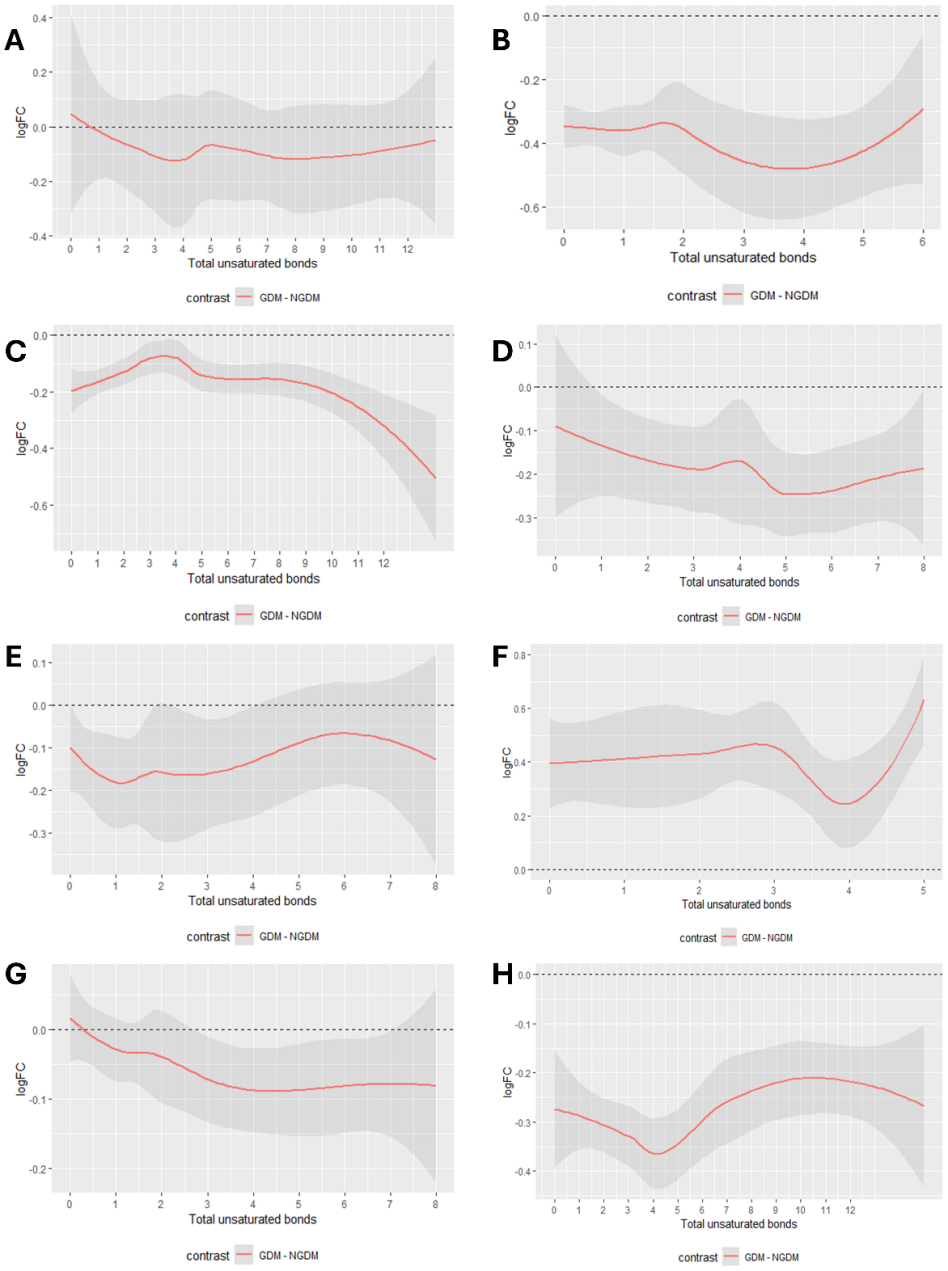
Analysis of unsaturation-logFC in lipids. (A) The unsaturation trend analysis plot of DG. (B) The unsaturation trend analysis plot of LPC. (C) The unsaturation trend analysis plot of PC. (D) The unsaturation trend analysis plot of PE. (E) The unsaturation trend analysis plot of PG. (F) The unsaturation trend analysis plot of PI. (G) The unsaturation trend analysis plot of SM. (H) The unsaturation trend analysis plot of TG.

In the analysis of lipid profiles between pregnant women with GDM and non-GDM controls, distinct trends in the logFC of lipid species were observed. Carbon chain length significantly influenced logFC values across lipid classes. Diglyceride (DG) and sphingomyelin (SM) exhibited an initial decline in logFC values followed by fluctuations with increasing carbon chain length (Figs. 6A, 6G). In contrast, LPC demonstrated a progressive increase in logFC values with chain elongation (Fig. 6B). Phosphatidylcholine (PC), PE, phosphatidylglycerol (PG), and TG reached peak or trough logFC values at specific chain lengths, followed by divergent trends (Figs. 6C, 6D, 6E, 6H). Phosphatidylinositol (PI) displayed a pronounced oscillatory logFC pattern across chain lengths (Fig. 6F). Regarding unsaturation, DG, LPC, and SM showed minimal logFC values at an unsaturation degree of 4 (Figs. 7A, 7B, 7G). The impact of unsaturation on PC, PE, and PG was more heterogeneous, with logFC values varying nonlinearly across unsaturation levels (Figs. 7C, 7D, 7E). PI and TG attained extremal logFC values at specific unsaturation states (Figs. 7F, 7H).

### Further identification of hub lipids

**Figure 8:**
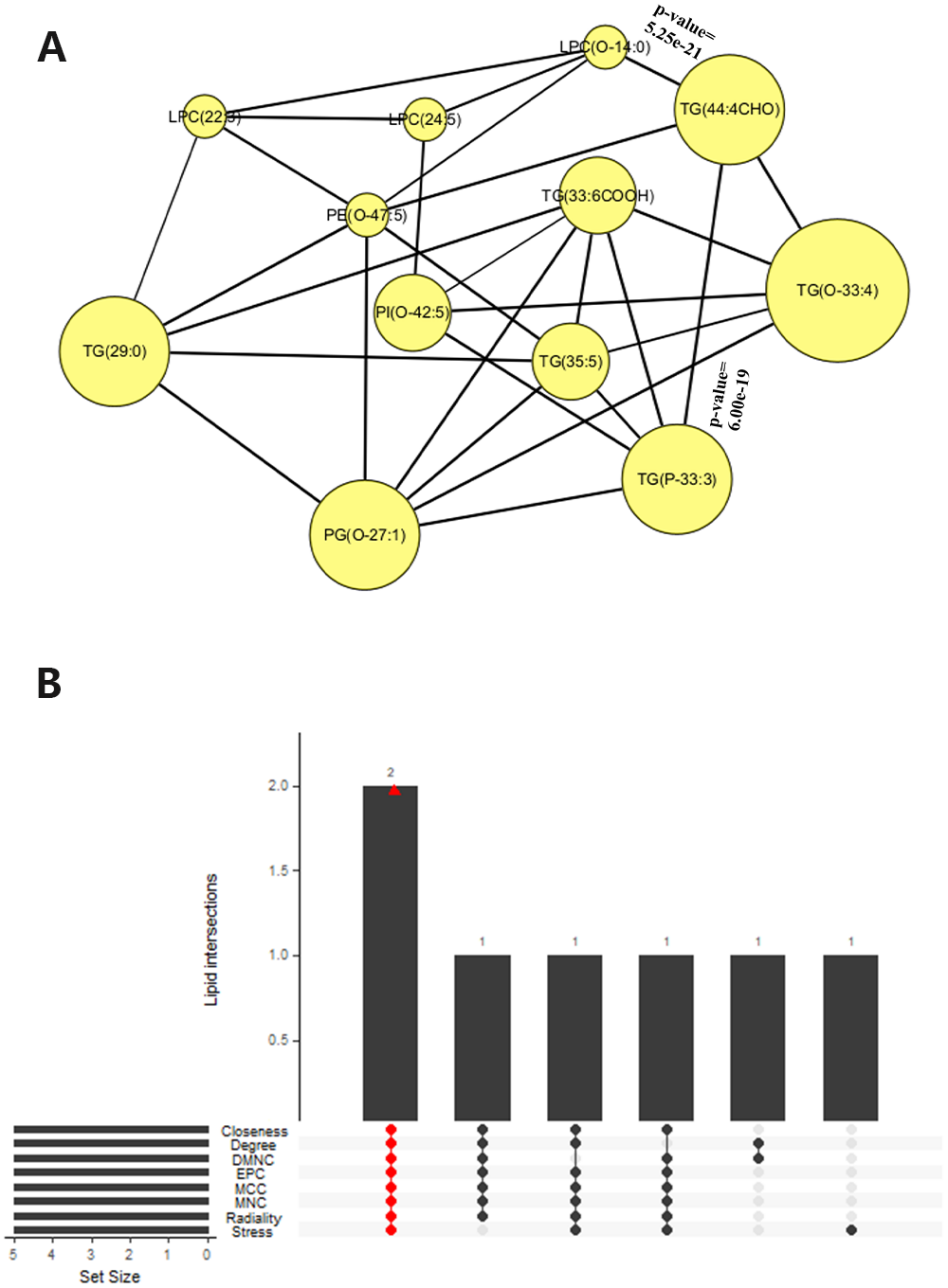
Further identification analysis of hub lipids. (A) In the correlation network graph, the nodes representing differential lipids, and the line thicknesses representing p-values of correlations between any two lipids (the two most statistically significant edges in the graph, those linking LPC(O-14:0) with TG(44:4CHO) and TG(44:4CHO) with TG(P-33:3), explicitly annotated with their corresponding p-values). (B) The set of every topological method containing 5 lipids in the visualization of Upset plot.

Figure 8A clearly visualized the correlations among the 12 differential lipids based on DSPC network. The hub lipids PG(O-27:1) and TG(35:5) were identified by the intersections of all sets from eight topological methods in the Upset plot (Fig. 8B).

### Development and assessment of biomarkers combination based on the GDM detection models

**Figure 9:**
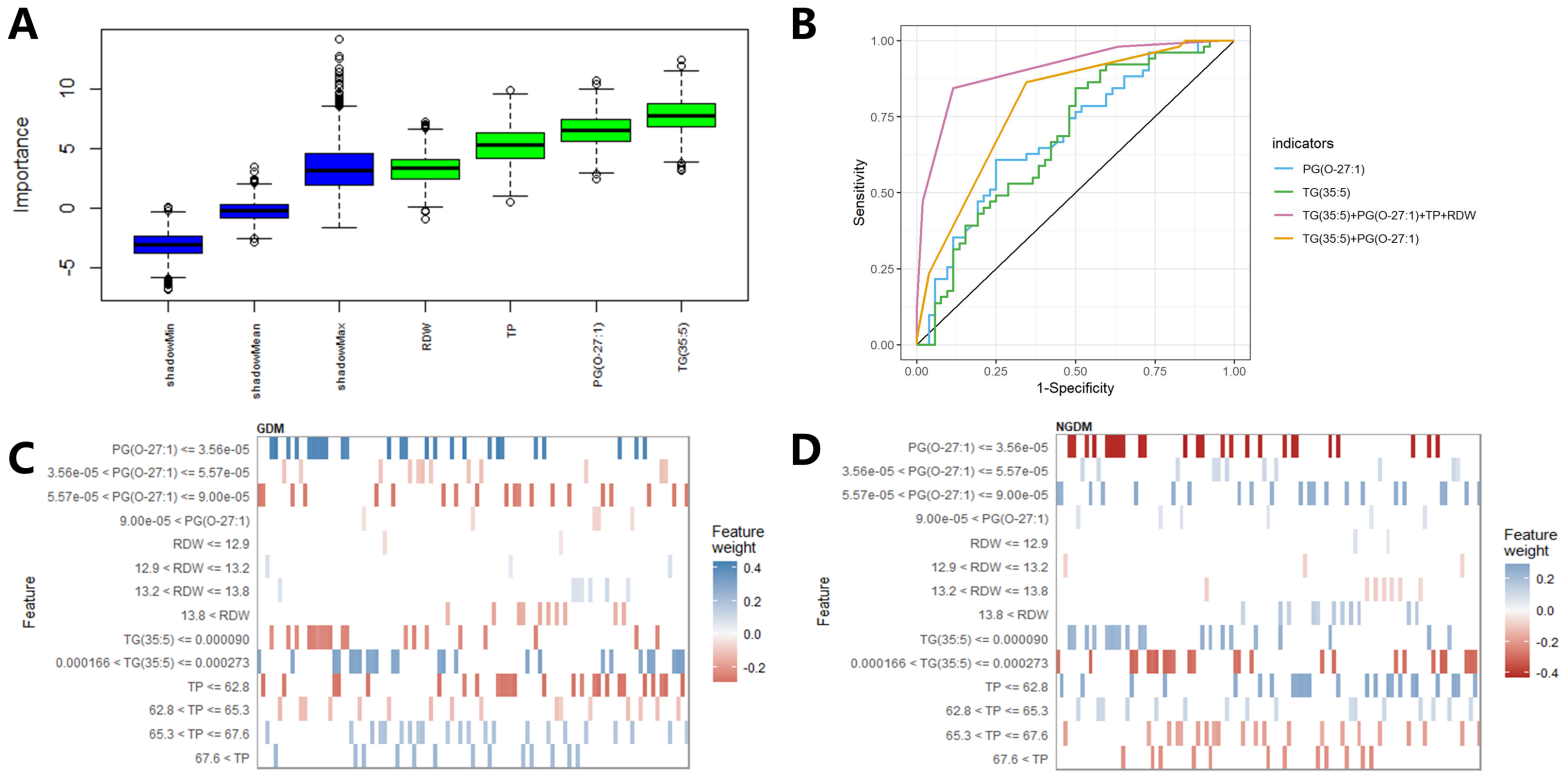
Establishment of the GDM detection model. (A) Boruta selection of four predictors with importance ranking. The attributes of the predictors represented by Z-score, with the blue and green boxes representing the Z-scores of the rejected and confirmed attributes, respectively. (B) ROC curves of the four settings (PG(O-27:1), TG(35:5), TG(35:5)+PG(O-27:1), TG(35:5)+PG(O-27:1)+TP+RDW). (C–D) Explanations of the GDM detection model based on LIME. The graphs of LIME showing how the important predictors contributed to predicting GDM/Non-GDM.

A total of four variables PG(O-27:1), TG(35:5), total protein (TP) and red blood cell distribution width (RDW) were screened by Boruta, TG(35:5) with the highest Z-score of importance (Fig. 9A). ROC curve analysis indicated that the performance of the combined PG(O-27:1), TG(35:5), TP, and RDW was the best among the four settings (Fig. 9B, Table 4). Furthermore, LIME elucidated the predictive role of these four variables in the classification of two outcomes (Figs. 9C, 9D). The criteria for tendency prediction of GDM in pregnant women were: PG(O-27:1) ≤ 3.56e−05, 12.9 < RDW ≤ 13.8, 0.000166 < TG(35:5) ≤ 0.000273 and 65.3 < TP. Conversely, non-GDM pregnant women were characterized by: 3.56e−05 < PG(O-27:1), 13.8 < RDW, TG(35:5) ≤ 0.00009 and TP ≤ 65.3.

**Table 4:** Clinical parameter and hub lipid analysis based on the ROC curves.

Settings	Cutoff value	Sensitivity	Specificity	Precision	AUC (95% CI)	Accuracy (95% CI)	Youden index
TG(35:5)	0.000242	0.843	0.500	0.623	0.649 (0.574–0.783)	0.670 (0.570–0.759)	0.343
PG(O-27:1)	0.0000467	0.608	0.750	0.705	0.687 (0.584–0.790)	0.680 (0.580–0.768)	0.358
TG(35:5)+						
PG(O-27:1)	–	0.863	0.654	0.710	0.790 (0.710–0.870)	0.757 (0.663–0.836)	0.517
TG(35:5)							
PG(O-27:1)+	–	0.843	0.885	0.878	0.904 (0.849–0.960)	0.864 (0.783–0.924)	0.728
TP+RDW							

**Notes.**

These ROC curve metrics encompassed cutoff value, specificity, sensitivity, precision, AUC (95% confidence interval (CI)), accuracy (95% CI) and Yoden index.

## Discussion

### Lipidome biological significance in pathogenesis of GDM

Prior work has established that lipid dyshomeostasis is not only a classic hallmark of GDM pathophysiology but also interlocks with glucose metabolic derangements in GDM (Eppel et al., 2021). Lipidomic analysis of GDM has shown that the pathways related to LPC and PI are highly significant. Most GDM patients are accompanied by chronic pancreatic functional dysfunction (Kc, Shakya & Zhang, 2015; Alejandro et al., 2020). The promoter methylation of ATP8b1 in pancreatic acinar cells (Lauber et al., 2003), leads to the downregulation of ATP8b1, reducing LPC levels, decreasing the clearance of apoptotic cells by macrophages (Xue et al., 2015), and potentially resulting in pancreatic fibrosis and structural changes (Yang et al., 2022), which can affect β-cell function and insulin secretion. This may indicate the lipid pathway linking LPC to GDM. PI participates in insulin signaling *via* the PI3K/AKT pathway, and abnormal PI expression can disrupt insulin and glucose metabolism, promoting the development of GDM (Feng et al., 2018; Zheng, Huang & Li, 2021). Therefore, PI may also have a potential link to the pathogenesis of GDM.

In our lipidomics enrichment analysis using the LION platform, two terms were identified as statistically significant: “fatty acids containing 3–5 double bonds” and “positive intrinsic curvature”. Previous studies have indicated that fatty acids with 3–5 double bonds belong to the category of polyunsaturated fatty acids (PUFAs), which can modulate the activity of nuclear receptors such as PPARγ, thereby influencing lipid metabolism and glucose homeostasis (Duttaroy & Basak, 2020). In the present study, on one hand, we further identified LPC(22:3) and LPC(24:5) in the blood of GDM patients as potential functional lipid molecules involved in this regulatory process. On the other hand, “positive intrinsic curvature”, which refers to membrane bending toward the intracellular side, generally reflects a state of dynamic cellular activity, signaling regulation (McMahon & Boucrot, 2011). It has previously been linked to lipid membrane structure and the development of insulin resistance (Petersen & Shulman, 2017). Alterations in membrane lipid composition during GDM may interfere with insulin signaling. In this study, lipids including LPC(22:3), LPC(24:5), LPC(O-14:0) and PI(O-42:5) were identified, and we hypothesize that they might contribute to GDM pathogenesis by modulating membrane curvature from the mechanistic angle that has received little attention to date. We observed decreased levels of both LPC and PI. LPC, a lipid known to promote positive curvature, is involved in fusion pore expansion during vesicle fusion, facilitating intercellular communication and signaling (Zhang & Jackson, 2010). The reduction in LPC may primarily impair insulin secretion by attenuating its inhibitory effect on endocytosis in pancreatic β-cells. This process may be mediated by genes such as SNARE family members (VAMP2, SNAP25), which are essential for vesicle fusion and insulin exocytosis (Zhu et al., 2012). By contrast, the decrease in PI may lead to reduced levels of PIP_2_ and PIP_3_ (Vanhaesebroeck & Alessi, 2000), thereby inhibiting GLUT4 vesicle trafficking and translocation in peripheral tissues and exacerbating insulin resistance. Moreover, under hyperglycemic conditions, PIP_2_ and PIP_3_ are more susceptible to degradation by phosphatases such as PTEN and SHIP, contributing to a vicious cycle of hyperglycemia (Rains & Jain, 2011).

To the best of our knowledge, this study identifies PG(O-27:1) and TG(35:5) as specific lipid species that may play a significant role in GDM. PG has a protective effect against oxidative damage, and its decrease may be related to pancreatic β-cell dysfunction and insulin gene suppression (Dong et al., 2024). Specifically, the expression of insulin genes and transcription factors such as PDX1 may be inhibited under conditions of oxidative stress, contributing to β-cell failure (Kaneto et al., 2005). Additionally, endoplasmic reticulum stress (ERS) is associated with the pathogenesis of GDM (Jawerbaum, 2016; Yung et al., 2016). TG, as an inducer of ERS, triggers the release of inflammatory mediators through the CHOP-PPARα-NF-κB signaling pathway, hindering glucose transport and thus affecting the occurrence and progression of GDM (Qiu et al., 2020; He et al., 2023). Notably, our study found that TG(35:5) was positively correlated with BMI (Deng et al., 2023), yet its levels were decreased in patients with GDM. However, other studies have reported that total TG levels are elevated in GDM, suggesting that different lipid species may exhibit distinct expression patterns in GDM (Zhang et al., 2024). Some LPC species also show a similar trend to total TG (Sharma et al., 2024; Hung et al., 2024). The potential mechanism underlying the reduced levels of TG(35:5) and LPC(22:3) in patients with GDM may partly be attributed to the selective uptake and metabolic preferences of the placenta for distinct fatty acids (Bitsanis et al., 2006).

Consequently, these two kinds of hub lipid species (PG(O-27:1) and TG(35:5)) biomarkers for predicting the risk of GDM. In this study, a carbon chain trend analysis and an exploration of the change in unsaturation degree were also conducted on PG and TG. It was found that both the carbon chain length and unsaturation degree exhibited complex patterns on the fluctuation of logFC.

### Association of lipid markers with maternal gestational status and offspring indices

The observed correlations between maternal lipid species and offspring developmental parameters suggest their potential roles in physiological regulation (Lackovic et al., 2024). In our cohort, PE(O-47:5), TG(O-33:4), and TG(P-33:3) were inversely associated with abdominal circumference, suggesting potential inhibitory effects on abdominal adipogenesis or related metabolic pathways. While the underlying mechanisms remain to be elucidated, such effects may involve the modulation of key adipogenic transcription factors, including PPARγ and C/EBPα, which are well-established regulators of adipocyte differentiation and lipid storage (Rosen & MacDougald, 2006). Similarly, negative correlations were observed between biparietal diameter and TG(P-33:3), TG(O-33:4), and PE(O-47:5), indicating that these lipid species may suppress cranial growth. This may involve the regulation of neural development genes such as SOX2 and BMP4, which exert effects on cranial bone and brain development (Wakamatsu & Uchikawa, 2021). Notably, while previous studies reported a positive correlation between maternal TG and fetal biparietal diameter in GDM (Song et al., 2023), our findings suggest that different lipid species may exert distinct functions. Thus, further precise stratification of lipid species is warranted when analyzing their associations with fetal imaging parameters. In late pregnancy, PG(O-27:1), TG(35:5), TG(O-33:4), and TG(P-33:3) were negatively correlated with amniotic fluid index, potentially through modulation of lipase activity in amniotic fluid, thereby influencing the fetal microenvironment (Merger et al., 1980). These findings provide new insights into lipid-mediated regulation of fetal organ development and metabolic homeostasis.

### Employing machine learning models for detection of GDM

In our study, the Boruta algorithm identified four determinant variables, specifically TG(35:5), PG(O-27:1), TP and RDW, which enabled high-accuracy detection of GDM using LogitBoost. According to the present results, the best GDM detection model (AUC = 0.904) showed better performance than existing models based on the metabolomic biomarkers and the proteomic biomarkers (Peng et al., 2022; Chen et al., 2023), though validating the fomer remains considerable.

It also surpassed models incorporating conventional clinical parameters such as fasting plasma glucose or insulin levels and genetic factors such as single nucleotide polymorphisms (Zhang et al., 2022; Li et al., 2024). By integrating multidimensional indicators, our model not only improves GDM prediction accuracy but also exhibits enhanced robustness and earlier risk identification capability.

Thus, LIME analysis based on these findings suggests that upon reaching specific thresholds, these biomarkers may be associated with an increased risk of GDM in pregnant women. The importance of TP and RDW within the model likely reflects their roles in systemic metabolism and hematologic status. For instance, elevated TP levels are often considered a marker of metabolic disturbance, conditions linked to the pathogenesis of GDM (Zeng et al., 2024). RDW, an indicator of erythrocyte size variation, is associated with microvascular dysfunction and inflammation, which are prevalent in diabetes mellitus, although the precise underlying mechanisms remain to be fully elucidated (Erdoğan et al., 2014). Therefore, these biomarkers hold the potential to inform risk stratification and early intervention strategies for the pregnant women. Nevertheless, their generalizability and diagnostic accuracy also need further validation in larger, independent cohorts.

## Conclusions

This study identified 12 serum lipid metabolites, derived from diverse lipid classes, that may be associated with the risk of GDM in the second trimester. They are primarily involved in TG and PG metabolic pathways. By integrating ML with bioinformatic approaches, the research established a lipidomic signature linked to GDM and provided a novel perspective on its underlying lipid metabolic mechanisms. Furthermore, the results of this study also require further confirmation in the future by utilizing more comprehensive data resources within the field, including the self-built external databases and comparable datasets from other research teams.

##  Supplemental Information

10.7717/peerj.20841/supp-1 Supplemental Information 1 R code for data analysis

10.7717/peerj.20841/supp-2 Supplemental Information 2 Clinical data

10.7717/peerj.20841/supp-3 Supplemental Information 3 Lipidomic data

10.7717/peerj.20841/supp-4 Supplemental Information 4 Codebook for clinical rawdata

## Additional Information and Declarations

### Data Availability

The following information was supplied regarding data availability: The code and data are available in the Supplemental Files.
