# Supplementary material for: Lipidomics and machine learning revealing dysregulation of specific triacylglycerol and phosphatidylglycerol as hub lipids associated with fetal growth in gestational diabetes mellitus
Source: PeerJ. 2026 Apr 7;14:e20841. doi: 10.7717/peerj.20841 (PMC13068011; doi:10.7717/peerj.20841)
Supplement: Supplemental Information 4 [file peerj-14-20841-s004.pdf]

CodeBook for clinical rawdata

The table of varable name, data type, description and coding.

Variable name	Data type	Description	Coding
ID	\	Designating identification number of the participants	\
Group	Categorical	The groups of the different participants in the study	"GDM" =group of participants with GDM;<br>"NGDM" =group of participants without GDM
Parity	Categorical	The number of born children of participants	"0"=none of existing child;<br>"1"=1 born child;<br>"2"=2 born children;<br>"3"=3 born children;<br>"4"= 4 born children
Neonatal_ weight	Numeric	Neonatal related indicator	\
Neonatal_ length	Numeric	Neonatal related indicator	\
Agpar_1min	Numeric	Neonatal related indicator (1-minute Apgar score)	\
Agpar_5min	Numeric	Neonatal related indicator (5-minute Apgar score)	\
Agpar_10min	Numeric	Neonatal related indicator (10-minute Apgar score)	\
Age	Numeric	The age of participants	\
Gestational_ weight_gain	Numeric	Pregnancy related indicator	\
Educated_ degree	Categorical	The educated degree of participants	"higher_education"= bachelor degree and above;<br>"secondary_ education"= secondary education;<br>"elementary_ education_and_ below"= elementary education and below
NT	Numeric	Fetal related indicator (Nuchal translucency)	\
CRL	Numeric	Fetal related indicator (Crown-	\

		rump length)	
ALT	Numeric	Clinical related indicator of participants	\
AST	Numeric	Clinical related indicator of participants	\
DRR	Numeric	Clinical related indicator of participants	\
TBIL	Numeric	Clinical related indicator of participants	\
DBIL	Numeric	Clinical related indicator of participants	\
IBIL	Numeric	Clinical related indicator of participants	\
TBA	Numeric	Clinical related indicator of participants	\
TP	Numeric	Clinical related indicator of participants	\
ALB	Numeric	Clinical related indicator of participants	\
GLO	Numeric	Clinical related indicator of participants	\
AGR	Numeric	Clinical related indicator of participants	\
ALP	Numeric	Clinical related indicator of participants	\
GGT	Numeric	Clinical related indicator of participants	\
CK	Numeric	Clinical related indicator of participants	\
CK_MB	Numeric	Clinical related indicator of participants	\
LDH	Numeric	Clinical related indicator of participants	\
K	Numeric	Clinical related indicator of participants	\
Na	Numeric	Clinical related indicator of participants	\
Cl	Numeric	Clinical related indicator of participants	\
Ca	Numeric	Clinical related indicator of participants	\
Mg	Numeric	Clinical related indicator of participants	\

P	Numeric	Clinical related indicator of participants	\
CO2	Numeric	Clinical related indicator of participants	\
GLU	Numeric	Clinical related indicator of participants	\
UREA	Numeric	Clinical related indicator of participants	\
CRE	Numeric	Clinical related indicator of participants	\
UA	Numeric	Clinical related indicator of participants	\
CHO	Numeric	Clinical related indicator of participants	\
TG	Numeric	Clinical related indicator of participants	\
HDLc	Numeric	Clinical related indicator of participants	\
LDLc	Numeric	Clinical related indicator of participants	\
apoA1	Numeric	Clinical related indicator of participants	\
apoB	Numeric	Clinical related indicator of participants	\
Sop	Numeric	Clinical related indicator of participants	\
CFR	Numeric	Clinical related indicator of participants	\
WBCC	Numeric	Clinical related indicator of participants	\
NE_per	Numeric	Clinical related indicator of participants	\
LY_per	Numeric	Clinical related indicator of participants	\
MO_per	Numeric	Clinical related indicator of participants	\
EO_per	Numeric	Clinical related indicator of participants	\
BA_per	Numeric	Clinical related indicator of participants	\
NE	Numeric	Clinical related indicator of participants	\
LY	Numeric	Clinical related indicator of participants	\

MO	Numeric	Clinical related indicator of participants	\
EO	Numeric	Clinical related indicator of participants	\
BA	Numeric	Clinical related indicator of participants	\
RBC	Numeric	Clinical related indicator of participants	\
HGB	Numeric	Clinical related indicator of participants	\
HCT	Numeric	Clinical related indicator of participants	\
MCV	Numeric	Clinical related indicator of participants	\
MCH	Numeric	Clinical related indicator of participants	\
MCHC	Numeric	Clinical related indicator of participants	\
RDW	Numeric	Clinical related indicator of participants	\
PLT	Numeric	Clinical related indicator of participants	\
PCT	Numeric	Clinical related indicator of participants	\
MPV	Numeric	Clinical related indicator of participants	\
PDW	Numeric	Clinical related indicator of participants	\
NRBC_per	Numeric	Clinical related indicator of participants	\
NRBC	Numeric	Clinical related indicator of participants	\
FT3	Numeric	Clinical related indicator of participants	\
FT4	Numeric	Clinical related indicator of participants	\
TSH3	Numeric	Clinical related indicator of participants	\
25OHD	Numeric	Clinical related indicator of participants	\
Fer	Numeric	Clinical related indicator of participants	\
PT	Numeric	Clinical related indicator of participants	\

INR	Numeric	Clinical related indicator of participants	\
APTT	Numeric	Clinical related indicator of participants	\
TT	Numeric	Clinical related indicator of participants	\
FIB	Numeric	Clinical related indicator of participants	\
PLGF	Numeric	Clinical related indicator of participants	\
sFLT1	Numeric	Clinical related indicator of participants	\
BPD_ST	Numeric	Indicators related fetal ultrasound examination (fetal biparietal diameter during second trimester)	\
HC_ST	Numeric	Indicators related fetal ultrasound examination (fetal head circumference during second trimester)	\
AC_ST	Numeric	Indicators related fetal ultrasound examination (fetal abdominal circumference during second trimester)	\
FL_ST	Numeric	Indicators related fetal ultrasound examination (fetal femur length during second trimester)	\
MVP_ST	Numeric	Indicators related fetal ultrasound examination (fetal maximum vertical pocket during second trimester)	\
AFI_ST	Numeric	Indicators related fetal ultrasound examination (fetal amniotic fluid index during second trimester)	\
PT_ST	Numeric	Indicators related fetal ultrasound examination (fetal placental thickness during second trimester)	\
PM_ST	Categorical	Indicators related fetal ultrasound examination (fetal placental maturity during second trimester)	“0”=Grade 0; “1”=Grade 1; “2”=Grade 2; “3”=Grade 3
BPD_TT	Numeric	Indicators related fetal ultrasound examination (fetal biparietal diameter during third trimester)	\
HC_TT	Numeric	Indicators related fetal ultrasound	\

		examination (fetal head circumference during third trimester)	
AC_TT	Numeric	Indicators related fetal ultrasound examination (fetal abdominal circumference during third trimester)	\
FL_TT	Numeric	Indicators related fetal ultrasound examination (fetal femur length during third trimester)	\
MVP_TT	Numeric	Indicators related fetal ultrasound examination (fetal maximum vertical pocket during third trimester)	\
AFI_TT	Numeric	Indicators related fetal ultrasound examination (fetal amniotic fluid index during third trimester)	\
PT_TT	Numeric	Indicators related fetal ultrasound examination (fetal placental thickness during third trimester)	\
PM_TT	Categorical	Indicators related fetal ultrasound examination (fetal placental maturity during third trimester)	"0"=Grade 0; "1"=Grade 1; "2"=Grade 2; "3"=Grade 3
PSQIscore	Numeric	Participants' score of Pittsburgh sleep quality index	\
EPDSscore	Numeric	Participants' score of Edinburgh Postnatal Depression Scale	\
